# Modulation of Intestinal Barrier and Bacterial Endotoxin Production Contributes to the Beneficial Effect of Nicotinic Acid on Alcohol-Induced Endotoxemia and Hepatic Inflammation in Rats

**DOI:** 10.3390/biom5042643

**Published:** 2015-10-16

**Authors:** Wei Zhong, Qiong Li, Wenliang Zhang, Qian Sun, Xinguo Sun, Zhanxiang Zhou

**Affiliations:** 1 Center for Translational Biomedical Research, University of North Carolina at Greensboro, North Carolina Research Campus, Kannapolis, NC 28081, USA; E-Mails: w_zhong@uncg.edu (W.Z.); liqiongbmu@gmail.com (Q.L.); w_zhang2@uncg.edu (W.Z.); q_sun@uncg.edu (Q.S.); x_sun4@uncg.edu (X.S.); 2 Department of Nutrition, University of North Carolina at Greensboro, North Carolina Research Campus, Kannapolis, NC 28081, USA

**Keywords:** alcohol, endotoxemia, inflammation, gut permeability, nicotinic acid

## Abstract

Alcohol consumption causes nicotinic acid deficiency. The present study was undertaken to determine whether dietary nicotinic acid supplementation provides beneficial effects on alcohol-induced endotoxin signaling and the possible mechanisms at the gut-liver axis. Male Sprague-Dawley rats were pair-fed the Lieber-DeCarli liquid diets containing ethanol or isocaloric maltose dextrin for eight weeks, with or without dietary supplementation with 750 mg/liter nicotinic acid. Chronic alcohol feeding elevated the plasma endotoxin level and activated hepatic endotoxin signaling cascade, which were attenuated by nicotinic acid supplementation. Alcohol consumption remarkably decreased the mRNA levels of claudin-1, claudin-5, and ZO-1 in the distal intestine, whereas nicotinic acid significantly up-regulated these genes. The concentrations of endotoxin, ethanol, and acetaldehyde in the intestinal contents were increased by alcohol exposure, and niacin supplementation reduced the intestinal endotoxin and acetaldehyde levels. Nicotinic acid supplementation upregulated the intestinal genes involved in aldehyde detoxification via transcriptional regulation. These results demonstrate that modulation of the intestinal barrier function and bacterial endotoxin production accounts for the inhibitory effects of nicotinic acid on alcohol-induced endotoxemia and hepatic inflammation.

## 1. Introduction

Alcohol abuse causes alcoholic liver disease (ALD), but the mechanisms of disease pathogenesis have not been well defined. Alcohol is metabolized mainly in the liver and the process of alcohol metabolism generates acetaldehyde and reactive oxygen species, which are critical mediators in the pathogenesis of ALD [1,2,3,4]. A clinical study found that intestinal permeability was elevated only in alcoholics with liver disease but in alcoholics without liver disease [5], which suggests that gut leakiness may be a key co-factor in triggering the development of ALD. Increasing evidence suggest that the elevation of blood endotoxin level, namely endotoxemia, plays a critical role in the pathogenesis of ALD [6,7,8]. Endotoxin in the liver can activate Kupffer cells to produce proinflammatory cytokines which, in turn, induces hepatic inflammation [9]. Clinical studies have shown a positive correlation between the blood endotoxin levels and hepatic cytokine levels as well as the severity of liver damage [10,11,12]. Moreover, neutralization of circulating endotoxin has shown to attenuate alcohol-induced liver injury [13], suggesting a cause-effect relationship between endotoxemia and the pathogenesis of ALD.

Endotoxin is derived from the cell wall of Gram-negative bacteria that inhabit in the lumen of the distal intestine. Only trace amounts of endotoxin can penetrate through the intestinal barrier into the circulation system at physiological conditions [14]. However, increased endotoxin penetration occurs at pathophysiological conditions with intestinal barrier disruption and/or bacterial endotoxin overproduction [15]. Both clinical and experimental studies have shown that alcohol consumption impairs the intestinal barrier and leads to gut leakiness, as indicated by increased penetration of macromolecule makers from the intestine into the blood [5,16,17,18]. Mechanistic studies demonstrated that the expressions of tight junction proteins, such as claudin-1, occludin, and ZO-1, are reduced after alcohol exposure [19]. On the other hand, the attribution of bacterial endotoxin production to endotoxemia has also been experimentally tested in animal models, including inhibition of Gram-negative bacteria with probiotics and sterilization of the intestine with antibiotics [20,21].

Alcohol consumption is frequently associated with malnutrition, and the deficiencies of water-soluble vitamins and minerals are among the major changes detected in patients with ALD [22]. Nicotinic acid, also known as niacin, is one of the naturally occurring B3 vitamins. Alcohol consumption has long been shown to cause niacin deficiency which leads to pellagra [23]. Moreover, alcohol metabolism disrupts the cellular redox status by increasing the ratio of NADH/NAD^+^ [1]. Since Vitamin B3 is the precursor of NAD^+^, nicotinic acid deficiency may worsen alcohol-induced redox imbalance. Indeed, our previous report showed that nicotinic acid supplementation attenuated alcoholic steatosis through increasing cellular NAD^+^ level [24]. The objective of the present study was to determine whether dietary nicotinic acid supplementation could alleviate alcohol-induced endotoxemia and endotoxin signaling in the liver. The possible mechanisms underlying the beneficial effects of nicotinic acid at the gut-liver axis were also examined.

## 2. Results

### 2.1. Nicotinic Acid Supplementation Prevents Alcohol-Induced Elevation of Blood Endotoxin Level and Hepatic Endotoxin Signaling

**Figure 1:**
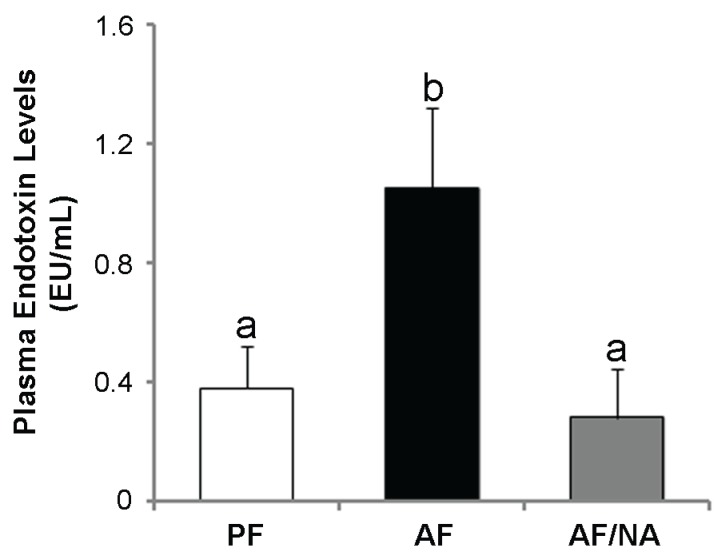
Plasma endotoxin levels in rats fed alcohol with or without nicotinic acid supplementation for 8 weeks. Endotoxin levels were measured by the limulus ameobocyte lysate method. Results are means ± SD (*n* = 6–8). Significant differences (*p* < 0.05, ANOVA) are identified by different letters. PF: pair-fed; AF: alcohol-fed; AF/NA: alcohol-fed with nicotinic acid supplementation.

**Figure 2:**
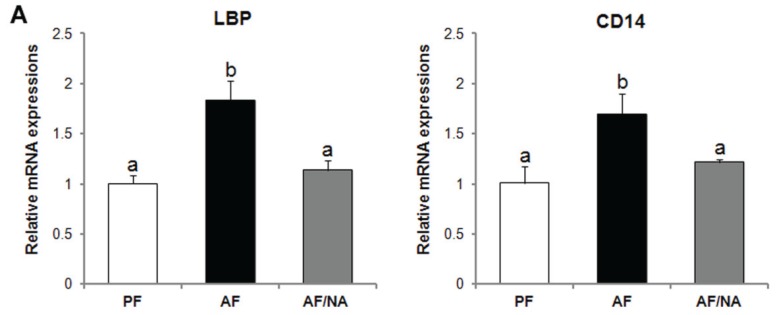

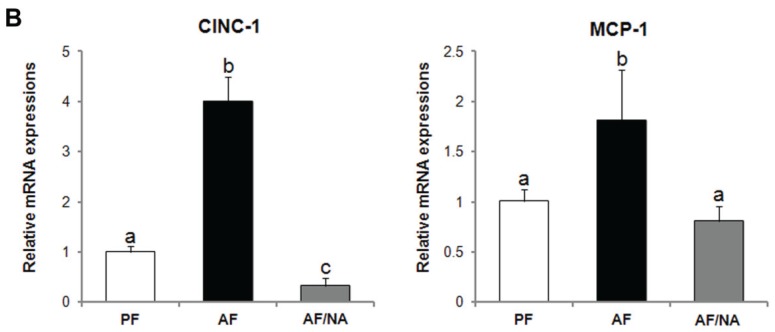
Hepatic gene expression of endotoxin signaling molecules (**A**) and cytokines (**B**) in rats fed alcohol with or without nicotinic acid supplementation for 8 weeks. Gene expression was assessed by qPCR assay of the mRNA level. Results are means ± SD (*n* = 6–8). Significant differences (*p* < 0.05, ANOVA) are identified by different letters. PF: pair-fed; AF: alcohol-fed; AF/NA: alcohol-fed with nicotinic acid supplementation; LBP: LPS binding protein.

**Figure 3:**
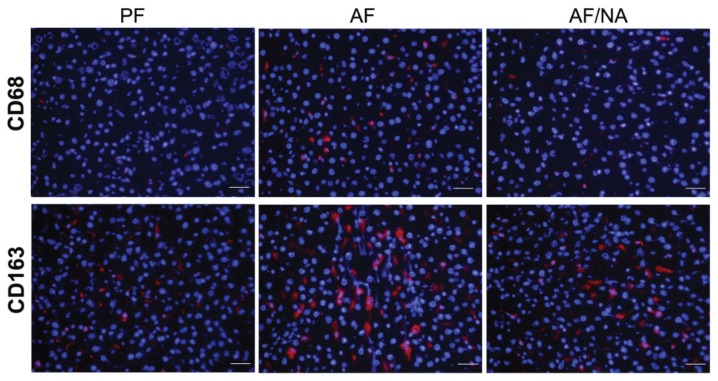
Immunofluorescence staining of hepatic CD68 and CD163 positive macrophages in rats fed alcohol with or without nicotinic acid supplementation for 8 weeks. Red color: CD68^+^ or CD163^+^ macrophages; blue color: 4',6-diamidino-2-phenylindole (DAPI) counterstaining of the nuclei. Scale bar: 20 μm. PF: pair-fed; AF: alcohol-fed; AF/NA: alcohol-fed with nicotinic acid supplementation.

Chronic alcohol feeding for eight weeks significantly increased the plasma endotoxin level compared to that of the PF rats, and dietary nicotinic acid supplementation prevented the elevation (Figure 1). In accordance, the hepatic mRNA levels of endotoxin signaling molecules LBP and CD14 (Figure 2A), and cytokines CINC-1 and MCP-1 (Figure 2B), were increased after chronic alcohol exposure. There was no significant difference of the TNF-α mRNA levels between the three groups. Dietary nicotinic acid supplementation to the AF mice normalized all these hepatic genes (Figure 2). Furthermore, immunohistochemical staining showed stronger fluorescence intensities of both the CD68^+^ and CD163^+^ macrophages in the liver of the AF rats, which was alleviated by dietary nicotinic acid supplementation (Figure 3).

### 2.2. Nicotinic Acid Supplementation Upregulates Intestinal Tight Junction Proteins and Reduces Intestinal Luminal Endotoxin Level

**Figure 4:**
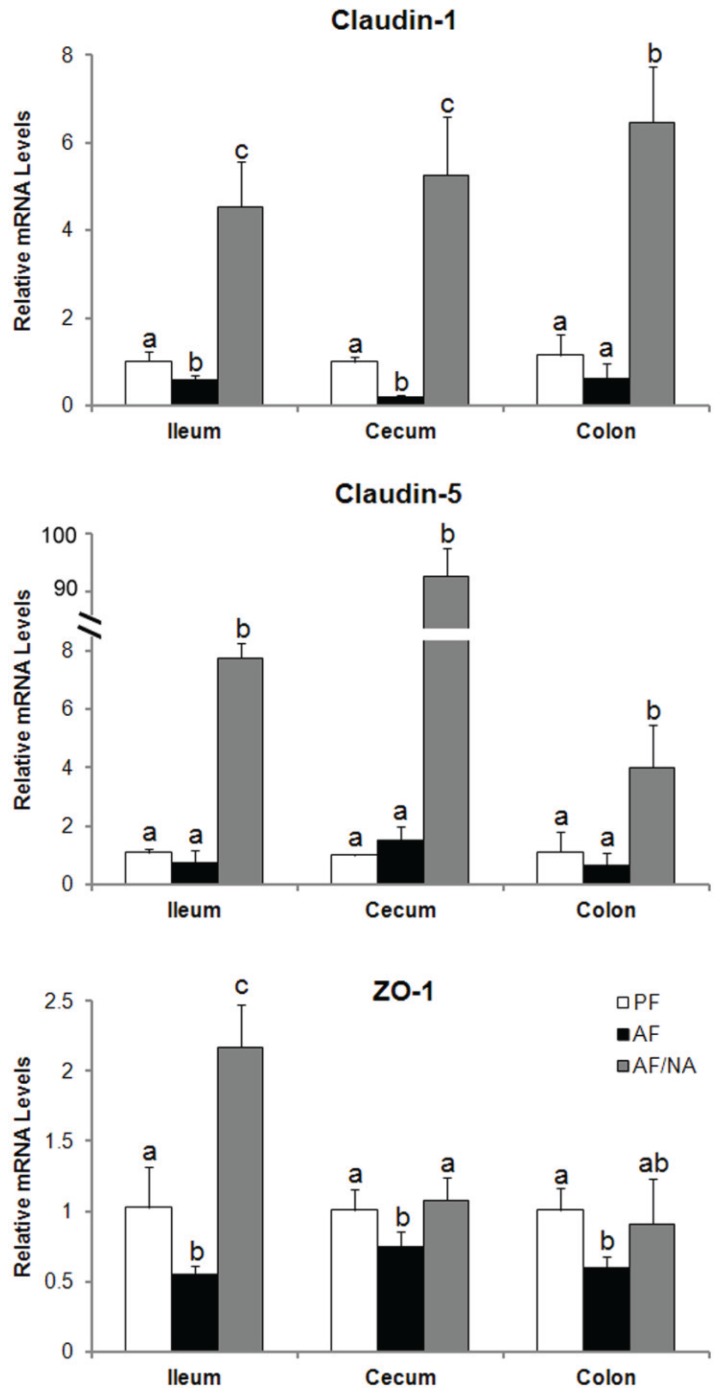
Expression of the intestinal tight junction proteins in rats fed alcohol with or without nicotinic acid supplementation for 8 weeks. Gene expression was assessed by qPCR assay of the mRNA level. Results are means ± SD (*n* = 6–8). Significant differences (*p* < 0.05, ANOVA) are identified by different letters. PF: pair-fed; AF: alcohol-fed; AF/NA: alcohol-fed with nicotinic acid supplementation.

**Figure 5:**
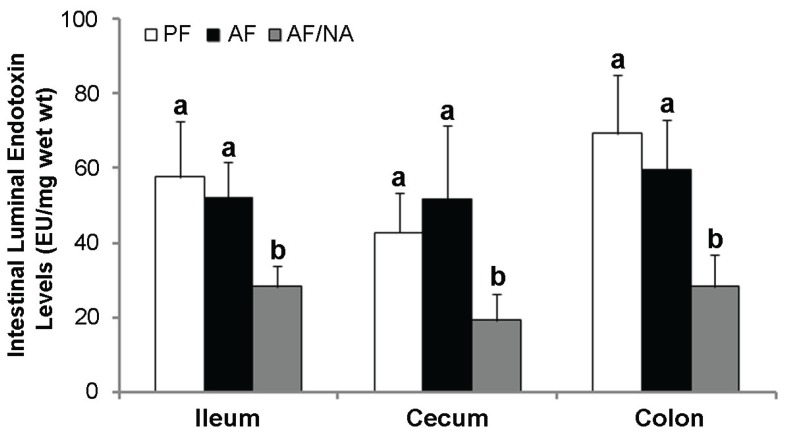
Intestinal luminal endotoxin levels in rats fed alcohol with or without nicotinic acid supplementation for 8 weeks. Endotoxin levels were measured by the limulus ameobocyte lysate method. Results are means ± SD (*n* = 6–8). Significant differences (*p* < 0.05, ANOVA) are identified by different letters. PF: pair-fed; AF: alcohol-fed; AF/NA: alcohol-fed with nicotinic acid supplementation.

To determine whether nicotinic acid prevents alcoholic endotoxemia through modulating the intestinal barrier and/or bacterial endotoxin production, intestinal tight junction proteins and luminal endotoxin level were analyzed. As shown in Figure 4, chronic alcohol feeding downregulated claudin-1 expression in the ileum and cecum, and decreased ZO-1 expression in all the three distal intestinal segments. Nicotinic acid supplementation dramatically upregulated the expression of claudin-1 and claudin-5, especially claudin-5, and normalized the expression of ZO-1. On the other hand, the intestinal luminal endotoxin levels of all the three distal intestinal segments were not affected by alcohol exposure, but were all significantly reduced by nicotinic acid in all the three intestinal segments (Figure 5).

**Figure 6:**
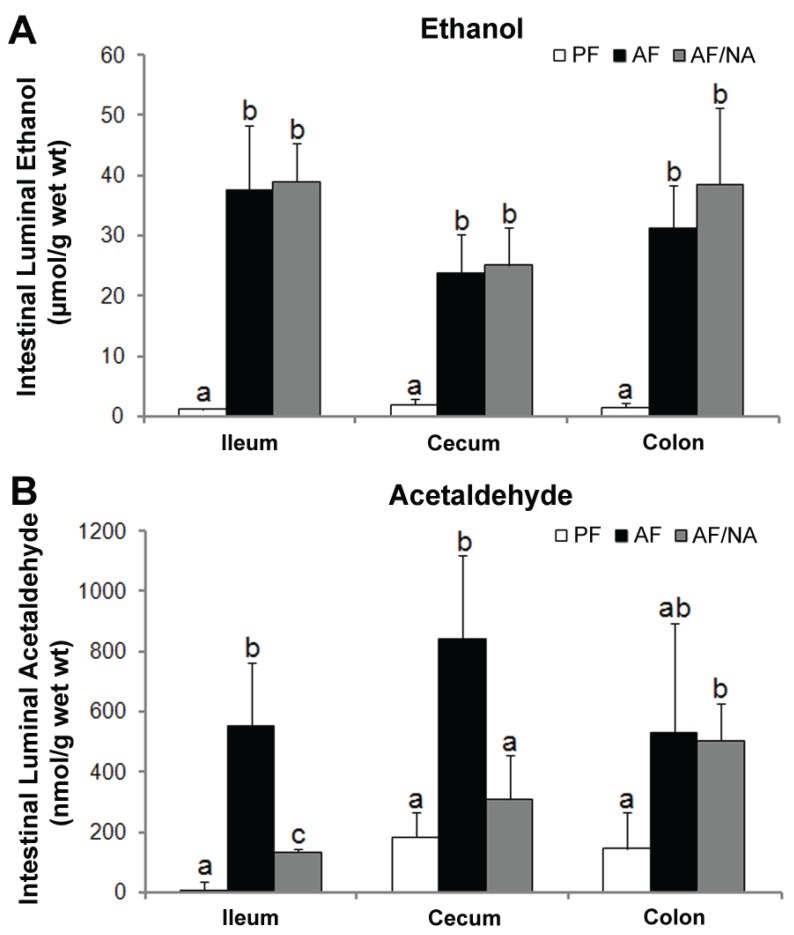
Intestinal luminal concentrations of ethanol (**A**) and acetaldehyde (**B**) in rats fed alcohol with or without nicotinic acid supplementation for 8 weeks. Ethanol and acetaldehyde in the ileal, cecal, and colonic lumen were measured by head-space GC-MS. Results are means ± SD (*n* = 6–8). Significant differences (*p* < 0.05, ANOVA) are identified by different letters. PF: pair-fed; AF: alcohol-fed; AF/NA: alcohol-fed with nicotinic acid supplementation.

### 2.3. Nicotinic Acid Supplementation Modulates Intestinal ALDH Genes and Reduces Intestinal Luminal Acetaldehyde Level

**Figure 7:**
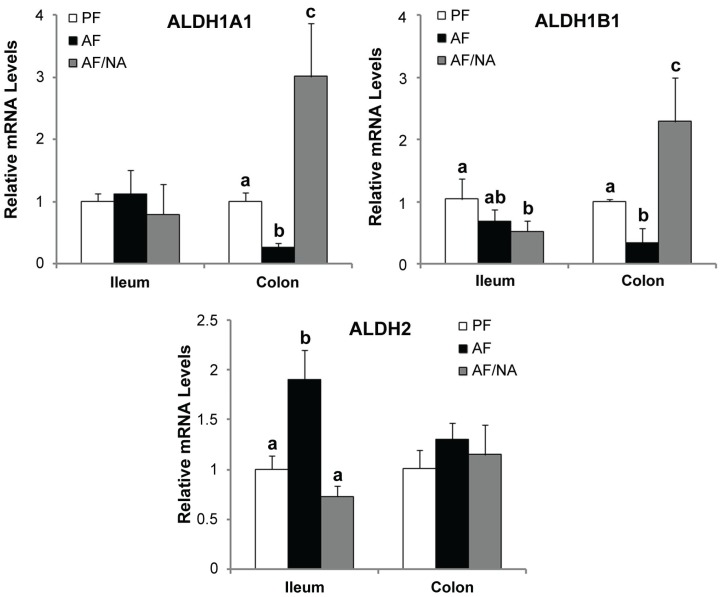
Expression of intestinal epithelial ALDH genes in rats fed alcohol with or without nicotinic acid supplementation for 8 weeks. Gene expression was assessed by qPCR assay of the mRNA levels. Results are means ± SD (*n* = 6–8). Significant differences (*p* < 0.05, ANOVA) are identified by different letters. PF: pair-fed; AF: alcohol-fed; AF/NA: alcohol-fed with nicotinic acid supplementation.

To determine whether nicotinic acid affects intestinal alcohol metabolism, ethanol and acetaldehyde concentrations within the intestinal lumen were measured. As shown in Figure 6A, ethanol concentration in the intestinal lumen was significantly increased in all the three distal intestinal segments, which was not affected by nicotinic acid supplementation. The acetaldehyde concentration in the intestinal lumen was increased in both the ileum and cecum in the AF rats compared to that of the PF controls; nicotinic acid supplementation significantly reduced the acetaldehyde level in the ileum and cecum without affecting the colon (Figure 6B). Moreover, to explore how nicotinic acid reduces the intestinal luminal acetaldehyde level, the expression of acetaldehyde metabolizing enzymes (ALDHs) was measured. As shown in Figure 7, chronic alcohol exposure downregulated Aldh1a1 and Aldh1b1 expression in the colon and upregulated Aldh2 expression in the ileum. Nicotinic acid upregulated Aldh1a1 and Aldh1b1 expression in the colon as compared to that of the AF rats.

### 2.4. Nicotinic Acid Supplementation Upregulates Intestinal HNF-1α and PPAR-α

**Figure 8:**
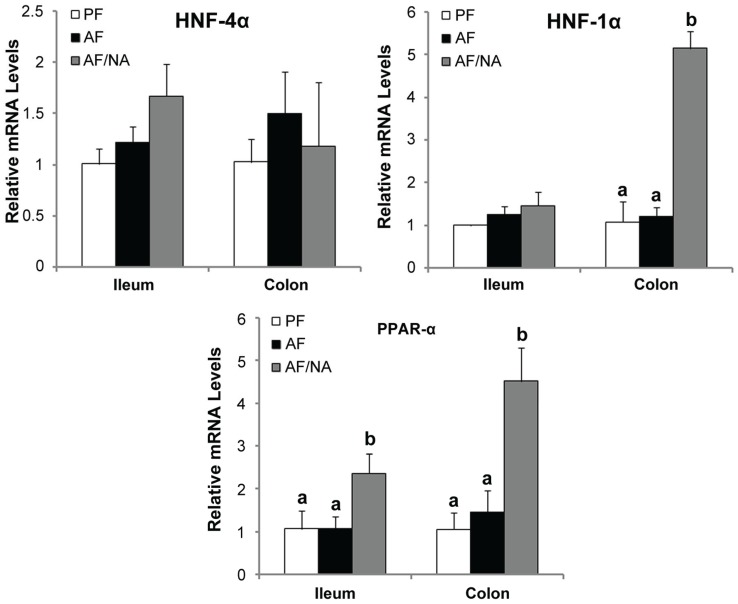
Expression of intestinal epithelial transcription factors in rats fed alcohol with or without nicotinic acid supplementation for 8 weeks. Gene expression was assessed by qPCR assay of mRNA levels. Results are means ± SD (*n* = 6–8). Significant differences (*p* < 0.05, ANOVA) are identified by different letters. PF: pair-fed; AF: alcohol-fed; AF/NA: alcohol-fed with nicotinic acid supplementation.

To understand the mechanism by which nicotinic acid modulates the intestinal barrier and ALDH expression, the expression of major intestinal transcription factors were measured. As shown in Figure 8, the expression of transcription factor Hnf-4α, Hnf-1α, and Ppar-α in the ileum and colon were not affected by chronic alcohol feeding. However, nicotinic acid supplementation upregulated Hnf-1α in the colon and Ppar-α in both the ileum and colon.

## 3. Discussion

The present study demonstrates that dietary nicotinic acid supplementation alleviated alcohol-induced endotoxemia and endotoxin signaling in the liver. The preventive effect of nicotinic acid on endotoxemia was associated with improved intestinal barrier and reduced bacterial endotoxin production. Another interesting finding is that nicotinic acid may modulate the intestinal acetaldehyde metabolism as indicated by reduced acetaldehyde level in the intestinal lumen and upregulated ALDH genes of the intestinal epithelium. Furthermore, upregulation of the intestinal transcription factors HNF-1α and PPAR-α likely mediates the beneficial effects of nicotinic acid on the intestine in response to alcohol intoxication.

Endotoxemia has been well documented in alcoholic patients [6,14,15]. Clinical studies have shown that the plasma endotoxin and hepatic cytokines including TNF-α, IL-6, and IL-8 were increased in patients with alcoholic hepatitis, while declined in the recovery phase [10]. To define the role of endotoxin in the pathogenesis of ALD, several strategies have been introduced to animal models, including inhibition of endotoxin producing bacteria with probiotics or antibiotics or neutralizing plasma endotoxin. Oral administration of *Lactobacillus* to the alcohol-fed rats significantly alleviated liver injury in association with an 83% decrease in the plasma endotoxin level [20]. Treatment with antibiotics, polymyxin B or neomycin, reduced nearly 75% of the plasma endotoxin level and completely prevented AST elevation [21]. A previous report from our group showed that neutralization of endotoxin by endotoxin neutralizing protein attenuated acute alcohol-induced TNF-α production and liver injury, indicating a cause-effect relationship between endotoxemia and hepatic cytokine expression as well as liver injury [13]. Several reports have also shown that prevention of endotoxemia correlated well with the inhibitory effects of medium-chain triglycerides or saturated fatty acids on proinflammatory cytokine production and liver injury [25,26,27]. The present study demonstrates that dietary nicotinic acid supplementation attenuated alcohol-induced endotoxemia and hepatic inflammation. This finding not only provides evidence in support of the role of endotoxin in the pathogenesis of ALD, but also suggests a potential therapeutic strategy for treating alcoholic endotoxemia and hepatitis.

The penetration of endotoxin from the intestinal lumen to the blood is tightly controlled by the intestinal barrier which may be disrupted at disease conditions, leading to increased intestinal permeability. The effects of alcohol exposure on impairing the intestinal barrier function have been repeatedly observed both in patients with ALD and in experimental ALD models [6,15]. Intestinal permeability to a variety of permeability markers, such as polyethyleneglycol, mannitol/lactulose, or ^51^Cr EDTA has been tested *in vivo* in ALD patients, and the intestinal permeability was significantly greater in alcoholics than in the normal subjects [5,16,17,18]. Similarly, increased permeability of the isolated gut sacs to macromolecules, such as HRP or FITC-dextran, has been repeatedly reported in rodent ALD models [19,28,29]. All these findings suggest that alcohol exposure impairs the function of the intestinal barrier. The barrier function of the intestinal epithelium is provided by paracellular apical junction complexes with tight junction being a central component [30,31]. Our previous reports have shown that alcohol exposure reduced the expression of tight junction proteins in association with elevation of plasma endotoxin level [19]. The present study found that the expressions of the intestinal tight junction proteins were reduced by alcohol exposure, and were upregulated by dietary nicotinic acid supplementation to a level that even higher than that of the control. Furthermore, HNF-1α and PPAR-α are critical intestinal transcription factors in the regulation of intestinal integrity and function [32,33], and upregulation of the two transcription factors may account for the molecular mechanism by which nicotinic acid provides beneficial effects on the intestinal barrier.

Quantitative (bacterial overgrowth) and qualitative (dysbiosis) changes of the GI microbiome have long been associated with liver diseases including ALD [34]. Disturbed gut microbiota homeostasis results in dysfunction of the intestinal barrier and translocation of bacteria and/or bacterial products, which eventually contribute to the progression of ALD. Alcohol consumption is well known to elicit bacterial overgrowth along the GI tract [35]. The number of both aerobic and anaerobic bacteria cultures of jejunal aspirates from alcoholic patients was distinctly higher than that from the control patients [36]. Similar trends were also observed in patients with alcoholic cirrhosis [37]. Bacterial overgrowth has also been documented in experimental models of ALD [38,39]. Bacteria, particularly the Gram-negative bacteria, produce endotoxins in the intestine. Therefore, bacterial overgrowth may attribute to the increased intestinal luminal endotoxin level and eventually endotoxemia. Even though intestinal bacterial overgrowth has been reported in alcoholic patients [30], whether or not the intestinal overproduction of endotoxin plays a crucial role in the development of alcoholic endotoxemia remains unclear.

Alcohol consumption not only results in quantitative changes of the intestinal microbiota, but also leads to an imbalance in the intestinal bacteria composition, namely enteric dysbiosis. Clinical studies have shown that patients with alcoholic cirrhosis had a lower proportion of *Bacteroidetes* and higher ones of *Proteobacteria* in the colon as compared to alcoholic patients without liver disease [40]. An animal study showed that chronic alcohol feeding for eight weeks caused a decline in the abundance of both *Bacteroidetes* and *Firmicutes* phyla, with a proportional increase in the Gram-negative *Proteobacteria* and Gram-positive *Actinobacteria* phyla in mice feces [39]. The *Proteobacteria* phylum includes Gram-negative bacteria, most of which are regarded as pathogenic species. Therefore, alcohol exposure-induced *Proteobacteria* expansion in the intestine strongly indicates a link between alcohol-induced alterations of gut microbiota and the elevated plasma endotoxin level and hepatic inflammation. Indeed, dietary supplementation with probiotics corrected alcohol-induced perturbation of the intestinal microbiota in association with reduced plasma endotoxin level [10]. These observations indicate that the inhibition of endotoxin-producing bacteria could be an important involving factor in alcoholic endotoxemia apart from the impaired intestinal barrier function. In the present study, dietary nicotinic acid supplementation reduced the endotoxin level in the intestinal lumen. The total level of intestinal luminal endotoxin may become a significant factor in the development of endotoxemia at a condition of impaired gut barrier. Thus, the alleviation of endotoxemia by nicotinic acid may involve two possible mechanisms, reducing intestinal endotoxin production and improving intestinal barrier function.

Alcohol consumption increases the level of acetaldehyde in the intestinal lumen. Increasing evidence suggest that acetaldehyde may play a crucial role in mediating alcohol-induced disruption of the intestinal barrier [41]. *In vitro* studies have shown that acetaldehyde disassembles the distribution of tight junction proteins including occludin and ZO-1 and dissociates these proteins from the actin cytoskeleton in Caco-2 cell monolayers [42,43]. The disassembly of tight junction molecules was also observed in human colonic biopsies treated with acetaldehyde *ex vivo* [44]. The present study demonstrated that dietary nicotinic acid significantly reduced the acetaldehyde level in distal intestinal lumen without affecting the intestinal luminal ethanol concentration. This finding suggests that reducing the intestinal luminal acetaldehyde level may attribute to the alleviation of alcohol-induced gut leakiness and endotoxemia. Moreover, the metabolism of endogenous and exogenous ethanol within the intestine is achieved by both the intestine and the microbiota. Experimental induction of bacteria overgrowth resulted in enhanced endogenous and/or exogenous ethanol metabolism and high concentrations of acetaldehyde both in the intestinal lumen and the portal blood [45,46]. Metronidazole increased the aerobic and anaerobic bacteria and led to high level of intracolonic acetaldehyde [47]. On the other hand, intracolonic acetaldehyde accumulation was prevented by antibiotic ciprofloxacin, which decreased colonic microbiota and fecal alcohol dehydrogenase activity [48]. However, the capacity of the intestinal mucosa and microbiota to metabolize acetaldehyde is rather low [49,50]. Although the present study showed that nicotinic acid upregulated some intestinal ALDH genes, the precise mechanism by which nicotinic acid reduces intestinal luminal acetaldehyde need further investigation.

## 4. Experimental Section

### 4.1. Animals and Alcohol Feeding Experiments

Male Sprague-Dawley rats were obtained from Charles River (Wilmington, MA, USA) and treated according to the experimental procedures approved by the Institutional Animal Care and Use Committee of the institution. Three-month-old rats were pair-fed with the liquid control diet (pair-fed group; PF), alcohol diet (alcohol-fed group; AF) or alcohol diet with nicotinic acid supplementation at 750 mg/L (AF/NA group), as described previously [24].

### 4.2. Endotoxin Assay

The endotoxin levels in the plasma and intestinal contents were assessed by a chromogenic kinetic limulus ameobocyte lysate (LAL) assay kit (Lonza, Walkersville, MD, USA) following the manufacture’s instruction. The concentration of endotoxin was expressed in endotoxin units (EU) per milliliter for plasma samples and EU per milligram weight of the intestinal contents for intestinal luminal samples.

### 4.3. qPCR Analysis

Total RNA was isolated from liver or ileum mucosa, and reverse transcribed with TaqMan Reverse Transcription Reagents (Applied Biosystems, Carlsbad, CA, USA). The gene expression of related mRNA was measured in triplicate by the comparative cycle threshold method using the 7500 real-time PCR system (Applied Biosystems). The primers sequences (Integrated DNA Technologies, Coralville, IA, USA) are shown in Table 1. The data were normalized to 18s rRNA mRNA levels and presented as fold changes, setting the values of PF rats as one.

### 4.4. Immunofluorescence Procedure

For detection of macrophages in the liver, cryostat sections of liver were incubated with anti-CD68 or anti-CD163 (BD Biosciences, San Jose, CA, USA) antibody overnight at 4 °C, respectively, followed by incubation with Alexa Fluor 594-conjugated donkey anti-mouse IgG (Jackson ImmunoResearch Laboratories, West Grove, PA, USA) for 30 min at room temperature. The nuclei were counterstained by 4',6-diamidino-2-phenylindole (DAPI; Life Technologies, Grand Island, NY, USA).

**Table 1:** Primer sequences used for qPCR analysis.

Gene	Genebank Accession No.	Forward Primer (5'–3')/Reverse Primer (5'–3')	Amplicon Size
**Aldh1a1**	NM_022407	GGGAAAGAGCCCTTGCATTGTGTT/	170 bp
		TGGCTCGCTCAACACTCTTTCTCA	
**Aldh1b1**	NM_001011975	AACAATACCAGGTACGGCTTGGCT/	163 bp
		TGCCATTGCCGGATTCCTTAAAGC	
**Aldh2**	NM_032416	ATTGGCTGATCTCATCGAACGGGA/	147 bp
		TACTTGTCAGCCCAGCCAGCATAA	
**Claudin-1/Cldn1**	NM_031699	AGGCAACCAGAGCCTTGATGGTAA/	83 bp
		CATGCACTTCATGCCAATGGTGGA	
**Claudin-5/Cldn5**	NM_031701	AATTCTGGGTCTGGTGCTGTGTCT/	102 bp
		ACGATATTGTGGTCCAGGAAGGCA	
**CINC-1/Cxcl1**	NM_030845	ACCCAAACCGAAGTCATAGCCAC/	181 bp
		ACTAGTGTTGTCAGAAGCCAGCGT	
**Hnf1α**	NM_012669	ACATTGAGCACAGAGGATGTGCCT/	105 bp
		GGAGCTGTCAGTGCGTTGTTGTTT	
**Hnf4α**	NM_022180	ATTCGGGCCAAGAAGATTGCCAAC/	108 bp
		AAGTTCACAGAAGGCCGGGATGTA	
**LBP/Lbp**	NM_017208	TGACATGTTACCGCCTGACTCCAA/	119 bp
		AGACCACTGTTCCAAGAAGCTCCA	
**MCP-1/Ccl2**	NM_031530	TGCTGTCTCAGCCAGATGCAGTTA/	131 bp
		TACAGCTTCTTTGGGACACCTGCT	
**Pparα**	NM_013196	AGCTCAGGACACAAGACGTTGTCA/	136 bp
		AGGGACTTTCCAGGTCATCTGCTT	
**Tnfα**	NM_012675	AGAACAGCAACTCCAGAACACCCT/	160 bp
		TGCCAGTTCCACATCTCGGATCAT	
**ZO-1/Tjp1**	NM_001106266	AAGATGGGATTCTTAGGCCCAGCA/	136 bp
		TCTTTGGCTGCAGGGCTATCTTCT	
**18s rRNA/Rn18s**	NR_046237	ACGGACCAGAGCGAAAGCAT/	152 bp
		TGTCAATCCTGTCCGTGTCC	

### 4.5. Ethanol and Acetaldehyde Assay

The concentrations of ethanol and acetaldehyde in the intestinal luminal contents were measured by headspace gas chromatography-mass spectrometry as described previously.

### 4.6. Statistical Analysis

All data are expressed as mean ± standard deviation (SD). The data were analyzed by analysis of variance (ANOVA) followed by Newman-Keuls’ *post hoc* test. Differences between groups were considered significant at *p* < 0.05.

## 5. Conclusions

In summary, the present study demonstrates that dietary nicotinic acid supplementation ameliorates alcoholic endotoxemia and hepatic inflammation, which provides novel experimental evidence that nicotinic acid possesses therapeutic potential in treating ALD. The protection of nicotinic acid against ALD is at least partially achieved through modulation of the intestinal barrier function and the intestinal bacteria.
